# Long-acting new generation antipsychotics in the maintenance treatment of bipolar disorders

**DOI:** 10.1192/j.eurpsy.2022.1026

**Published:** 2022-09-01

**Authors:** E. Yavuz, K. Altınbaş

**Affiliations:** Selcuk University Faculty of Medicine, Psychiatry, Konya, Turkey

**Keywords:** Long-acting injectable antipsychotics, maintenance, bipolar disorders, Relapse

## Abstract

**Introduction:**

Maintaining remission, preventing from future episodes, better treatment adherence and improving the quality of life are main aims of long-term treatment in bipolar disorders (BD). In recent years, new generation long-acting injectable (LAI) antipsychotics have been frequently used in maintenance treatment for bipolar disorders.

**Objectives:**

We aimed to review socio-demographic and clinical characteristics of bipolar patients taking LAI treatment for maintenance treatment.

**Methods:**

Clinical records of 44 bipolar patients who are on LAI treatment and followed in Mazhar Osman Mood Clinic (MOMC) of Selcuk University Medical Faculty were evaluated.

**Results:**

Nearly half of the patients were male (n:24, 54%). 43,2% of the patients were married. The mean age was 36.6±11.9 years and the mean duration of education was 11.5±3.9 years. All of the patients were diagnosed with bipolar 1 disorder. Most of the patients (65.9%) was on aripiprazole LAI while remaining was receiving paliperidone LAI for maintenance treatment. Ten of the patients discontinued the treatment due to the side effects and extrapyramidal side effects was the most common side effect. Relapse was observed in 25% of the patients and there was no difference between aripiprazole and paliperidone in terms of relapse rate.

**Conclusions:**

LAI new generation antipsychotics are taking place in long-term treatment of bipolar disorder via improving treatment adherence. Side effect profile of aripiprazole and paliperidone are different. However, we could not find any difference between two drugs in terms of side effects and relapse rates. Small sample size and shorter duration of follow-up should be considered as limitations.

**Disclosure:**

No significant relationships.

